# Correction: Li et al. Histone Deacetylases as Epigenetic Targets for Treating Parkinson’s Disease. *Brain Sci.* 2022, *12*, 672

**DOI:** 10.3390/brainsci15040335

**Published:** 2025-03-24

**Authors:** Yan Li, Zhicheng Gu, Shuxian Lin, Lei Chen, Valentina Dzreyan, Moez Eid, Svetlana Demyanenko, Bin He

**Affiliations:** 1 State Key Laboratory of Functions and Applications of Medicinal Plants, Engineering Research Center for the Development and Application of Ethnic Medicine and TCM (Ministry of Education), Guizhou Provincial Key Laboratory of Pharmaceutics, School of Pharmacy, School of Basic Medical Science, Guizhou Medical University, Guiyang 550004, China; yanli@gmc.edu.cn (Y.L.); guzhicheng0520@163.com (Z.G.); linshuxian365@163.com (S.L.); leichen@gmc.edu.cn (L.C.); 2 Laboratory of Molecular Neurobiology, Academy of Biology and Biotechnology, Southern Federal University, Stachki Ave. 194/1, 344090 Rostov-on-Don, Russia; dzreyan@sfedu.ru (V.D.); moez1995.mae@gmail.com (M.E.)

**Text Correction**

In the original publication [1], a correction has been made to Section 2, the last paragraph, the fourth sentence. The updated sentence is as follows:

On the contrary, although SIRT2 can directly bind with and deacetylate FOXO3a upon caloric restriction and oxidative stress.

**Citation updated**

The original citation [80] has been removed. With this correction, the order of some references has been adjusted accordingly.

**References**

Original reference [81] has been replaced with the following reference:

81. Wang, F.; Nguyen, M.; Qin, F.X.; Tong, Q. SIRT2 deacetylates FOXO3a in response to oxidative stress and caloric restriction. *Aging Cell* **2007**, *6*, 505–514.

The authors state that the scientific conclusions are unaffected. These correction were approved by the Academic Editor. The original publication has also been updated.
